
JOURNAL INFORMATION
==============================
NLM Title Abbreviation: Daniels Tex Med J
NLM Journal ID: 0006376

Daniel's Texas Medical Journal

ISSN: 0892-8487
Publisher: Biomedical Journal Digitization Project

ARTICLE INFORMATION
==============================
PMCID: PMC9176537
Article version: 1
Subjects: Book Notices

A Practical Manual of Diseases of the Skin

Print publication date: 1892 Oct
Volume: 8
Issue: 4
First page: 162
Last page: 162
License: This work is free of known copyright restrictions. For more information, please see PMC Back Issue Digitization.
License URL: https://creativecommons.org/publicdomain/mark/1.0/

==============================

USES OF WATER IN MODERN MEDICINE, Vol. 1, Leisure Library Series; by Simon Baruch, M. D. Paper back. Pp. 115. Price 25 cents. Geo. S. Davis, publisher, Detroit, Mich. 1892.

This little book does not attempt to bring water forth as a "cure all," but endeavors to give it its legitimate place in therapeutics, an object indeed worthy the best efforts of the able author and for which the American profession ought to exhibit gratitude in a substantial manner. B.

---

A PRACTICAL MANUAL OF DISEASES OF THE SKIN, By Geo. H. Rohe, M. D., Baltimore, assisted by J. Williams Lord, A. B., M. D., pp. 303. Price $1 net. For sale by the F. A. Davis Co., publishers, Philadelphia, 1892.

A caieful examination of this little book has convinced the writer that it is one of the best arranged and most practical of the small volumes on skin diseases now out. B.

---

### Publisher's Notes.

See new advertisement of Fairchild Bros. & Foster.

---

The Spectre of Inanition; See ad. of Arlington Chemical Company.

---

New Medical Books for Sale.—(Those you see reviewed each month.)

---

A pepsin that will digest 6000 times its own weight of coagulated egg-albumen is now manufactured by Sharp & Dohme. They send samples free on application. See advertisement on front cover.

---

A Convention of representatives of Southern Medical Colleges will be held in Louisville, Ky., November 16, 1892, for the purpose of considering the question of a higher standard of medical education.

---

Students who wish to complete their studies and graduate can do so by attending a good summer school. The medical department of the Wooster University, Cleveland, O., offers superior advantages. See ad.

---

The celebrated electrical and optical apparatus manufacturers, McIntosh Battery and Optical Co., have a full page advertisement in this issue. 'Twere like painting the lily to puff a house so famous for the superiority of their wares. See ad.
